# Altered Right Ventricular Mechanical Properties Are Afterload Dependent in a Rodent Model of Bronchopulmonary Dysplasia

**DOI:** 10.3389/fphys.2017.00840

**Published:** 2017-10-25

**Authors:** Jitandrakumar R. Patel, Gregory P. Barton, Rudolf K. Braun, Kara N. Goss, Kristin Haraldsdottir, Alexandria Hopp, Gary Diffee, Timothy A. Hacker, Richard L. Moss, Marlowe W. Eldridge

**Affiliations:** ^1^Department of Cell and Regenerative Biology, University of Wisconsin-Madison, Madison, WI, United States; ^2^Department of Pediatrics, University of Wisconsin-Madison, Madison, WI, United States; ^3^Department of Kinesiology, University of Wisconsin-Madison, Madison, WI, United States; ^4^Cardiovascular Research Center, University of Wisconsin School of Medicine and Public Health, Madison, WI, United States

**Keywords:** hyperoxia, right ventricle, pulmonary hypertension, myocardial contractility, troponin isoforms; myosin heavy chain isoforms

## Abstract

Infants born premature are at increased risk for development of bronchopulmonary dysplasia (BPD), pulmonary hypertension (PH), and ultimately right ventricular (RV) dysfunction, which together carry a high risk of neonatal mortality. However, the role alveolar simplification and abnormal pulmonary microvascular development in BPD affects RV contractile properties is unknown. We used a rat model of BPD to examine the effect of hyperoxia-induced PH on RV contractile properties. We measured *in vivo* RV pressure as well as passive force, maximum Ca^2+^ activated force, calcium sensitivity of force (pCa_50_) and rate of force redevelopment (*k*_tr_) in RV skinned trabeculae isolated from hearts of 21-and 35-day old rats pre-exposed to 21% oxygen (normoxia) or 85% oxygen (hyperoxia) for 14 days after birth. Systolic and diastolic RV pressure were significantly higher at day 21 in hyperoxia exposed rats compared to normoxia control rats, but normalized by 35 days of age. Passive force, maximum Ca^2+^ activated force, and calcium sensitivity of force were elevated and cross-bridge cycling kinetics depressed in 21-day old hyperoxic trabeculae, whereas no differences between normoxic and hyperoxic trabeculae were seen at 35 days. Myofibrillar protein analysis revealed that 21-day old hyperoxic trabeculae had increased levels of beta-myosin heavy chain (β-MHC), atrial myosin light chain 1 (aMLC1; often referred to as essential light chain), and slow skeletal troponin I (ssTnI) compared to age matched normoxic trabeculae. On the other hand, 35-day old normoxic and hyperoxic trabeculae expressed similar level of α- and β-MHC, ventricular MLC1 and predominantly cTnI. These results suggest that neonatal exposure to hyperoxia increases RV afterload and affect both the steady state and dynamic contractile properties of the RV, likely as a result of hyperoxia-induced expression of β-MHC, delayed transition of slow skeletal TnI to cardiac TnI, and expression of atrial MLC1. These hyperoxia-induced changes in contractile properties are reversible and accompany the resolution of PH with further developmental age, underscoring the importance of reducing RV afterload to allow for normalization of RV function in both animal models and humans with BPD.

## Introduction

Infants born prematurely are at increased risk for a number of comorbidities, including the development of chronic lung disease of prematurity, or bronchopulmonary dysplasia (BPD). After preterm birth, these infants generally require resuscitation, and are often supplemented with life-sustaining oxygen therapy for prolonged periods. Exposure to a relative hyperoxic environment at a time when infants should still be *in utero* in a hypoxic environment has been associated with perturbed development and has long-term consequences (Jobe and Bancalari, 2001). BPD is characterized by fewer and enlarged alveoli, increased lung collagen, blunted proliferation of arterioles, increased vascular tone, decreased vascular surface area, and thickening of arterial walls (Thibeault et al., 2003; Kaarteenaho-Wiik et al., 2004; Berkelhamer et al., 2013). The development of overt pulmonary hypertension (PH) results in increased afterload to the right ventricle (RV), leading to RV hypertrophy (RVH) and ultimately failure, with high infant morbidity and mortality (Bhat et al., 2012). Rodent models of BPD, characterized by postnatal hyperoxia exposure, recapitulate many of the findings of human disease, including arrested alveolar and vascular development, PH and RV dysfunction (Goss et al., 2015, 2017; Dumas de la Roque et al., 2017; Liang et al., 2017).

Recently, a study determined the effects of neonatal hyperoxia exposure on RV function in mice, demonstrating that 14-day old mice develops RVH and elevated phosphodiesterase 5 (PDE5) expression and activity, and these hyperoxia-induced changes were reversible by day 56 (Heilman et al., 2015). Indeed, previous report (Joshi et al., 2014) suggests that in later stages of childhood RV function and pulmonary arterial pressure is not different in children born preterm with existing chronic lung disease compared to term-born children who do not have chronic lung disease. However, there is limited, if any, information regarding the effects of neonatal exposure to hyperoxia on contractile properties and myofibrillar protein expression in the rodent RV. Thus, our aim was to use similar age groups of rats pre-exposed to 14 days of postnatal hyperoxia as Heilman et al. to investigate the effects of hyperoxia on *in vivo* RV pressure and cellular contractile properties and myofibrillar protein expression in RV. However, we chose to use 21 and 35 day old rats to investigate the effects of hyperoxia on *in vivo* RV pressure and cellular contractile properties and myofibrillar protein expression in RV based on our preliminary experiments, in which we were unable to isolate useable trabeculae for mechanical measurements from 14 days old rats and found mechanical properties of 35-day hyperoxic rats to be similar to aged matched normoxic rats. We hypothesized that postnatal hyperoxia exposure in rats would result in altered RV function which will correspond with PH.

## Methods and materials

### Animals

Timed pregnant Sprague-Dawley dams (Envigo, Indianapolis, IN) were allowed to deliver naturally at term in house. Irrespective of sex, the newborn pups were divided into two groups within 12 h of birth: (1) room air (normoxic), and (2) 14-day hyperoxia (hyperoxic). Both groups were housed in standard cages within a 30″ × 20″ × 20″ polypropylene chamber with a clear acrylic door. Oxygen concentration within the hyperoxia chamber was maintained at a fraction of inspired oxygen of 0.85 ± 0.03 using a continuous oxygen sensor, while the normoxia chamber was maintained at 0.21. Dams were rotated between room air and hyperoxia every 24 h to prevent oxygen-induced maternal toxicity. After 14 days, hyperoxic pups were returned to room air. Pups were weaned at 24 days. The UW School of Medicine and Public Health Animal Care and Use Committee approved all procedures involving animal care and handling.

### Invasive RV pressure

RV pressure measurements were performed at the University of Wisconsin Cardiovascular Physiology Core, as previously described (Hacker et al., 2006; Tabima et al., 2010). Briefly, 21 and 35-day-old rats were anesthetized with urethane (1.2 g/kg via intraperitoneal injection), orally intubated, and mechanically ventilated (Harvard Apparatus). The chest cavity was entered through the sternum and the chest wall and lungs were gently retracted to expose the RV. A 1.9F variable segment length admittance pressure catheter (Scisense, London, Ontario, Canada) was introduced into the RV using a 24-gauge needle. The magnitude and phase of the electrical E3 admittance and RV pressure were continuously recorded and analyzed using commercial software (Notocord Systems, Croissy Sur Seine, France).

### Preparation of skinned trabeculae

Right ventricular trabeculae were isolated as described previously (Olsson et al., 2004; Patel et al., 2012). Briefly, the hearts were dissected from 21- and 35-day old normoxic and hyperoxic rats anesthetized with inhaled isoflurane. The hearts were then pinned down to the base of a dissecting dish filled with modified Ringer's solution (in mmol/L: NaCl, 120; NaHCO_3_, 19; Na_2_HPO_4_, 1.2; MgSO_4_, 1.2; KCl, 5; CaCl_2_, 1; glucose, 10; pH 7.4; ~22°C) pre-equilibrated with 95% O_2_/5% CO_2_. After the right ventricles were cut open, the hearts were exposed to fresh Ringer's solution containing 20 mM 2,3-butanedione monoxime (BDM) for 30 min (2x solution change). BDM was used to protect the trabeculae from cell contracture and muscle damage during dissection (Mulieri et al., 1989). Right ventricular trabeculae were then dissected free, tied to wooden applicator sticks to hold muscle length fixed, and transferred to relaxing solution (in mmol/L: 100 KCl, 20 imidazole, 7 MgCl_2_, 2 EGTA, and 4 ATP; pH 7.0; 4°C) containing 1% Triton X-100. Following overnight skinning, the trabeculae were washed in fresh relaxing solution (~1 h) and then stored at −20°C in relaxing solution containing glycerol (50:50 v/v). The skinned trabeculae were used in experiments within 1 week.

### Experimental solutions, apparatus, and protocols

Solutions composition used for mechanical measurements were calculated using the computer program of Fabiato (1988) and the stability constants (corrected to pH 7.0 and 22°C) listed by Godt and Lindley (1982). Both pCa 9.0 and pre-activating solution contained (in mmol/L) 100 BES, 15 creatine phosphate, 4.66 ATP and 5 DTT. In addition, pCa 9.0 solution contained (in mmol/L) 7 EGTA, 0.02 CaCl_2_, 5.49 MgCl_2_, and 63.14 potassium propionate, whereas pre-activating solution contained (in mmol/L) 0.07 EGTA, 5.29 MgCl_2_, and 84.01 potassium propionate. pCa 4.5 solution contained (in mmol/L) 100 BES, 15 creatine phosphate, 4.72 ATP, 7 EGTA, 7.01 CaCl_2_, 5.29 MgCl_2_, 5 DTT, and 49.61 potassium propionate. pH of all solutions was adjusted to 7.0 with KOH. A range of Ca^2+^ activating solutions (pCa 6.2 to 5.4) were prepared by mixing solutions of pCa 9.0 and pCa 4.5.

On the day of an experiment, skinned trabeculae were incubated in relaxing solution for 30 min before cutting them free from the sticks and trimming their ends. The trimmed trabeculae were then transferred to a stainless steel experimental chamber containing pCa 9.0 solution (Moss et al., 1983). The ends of each trabecula were tied to the arms glued to a motor (model 312B, Aurora Scientific) and a force transducer (model 403; Aurora Scientific), as previously described (Moss et al., 1983). The chamber assembly was then placed on the stage of an inverted microscope (Olympus) fitted with a 40 × objective and a CCTV camera (model WV-BL600, Panasonic). The light from a halogen lamp was used to illuminate the skinned preparations. Bitmap images of the preparations were acquired using an AGP 4X/2X graphics card and associated software (ATI Technologies) and were used to assess mean sarcomere length (SL) during the course of each experiment. Changes in force and motor position were sampled (16-bit resolution, DAP5216a, Microstar Laboratories) at 2.0 kHz using SLControl software developed in this laboratory (http://www.slcontrol.com). Data were saved to computer files for later analysis.

**Figure 1:**
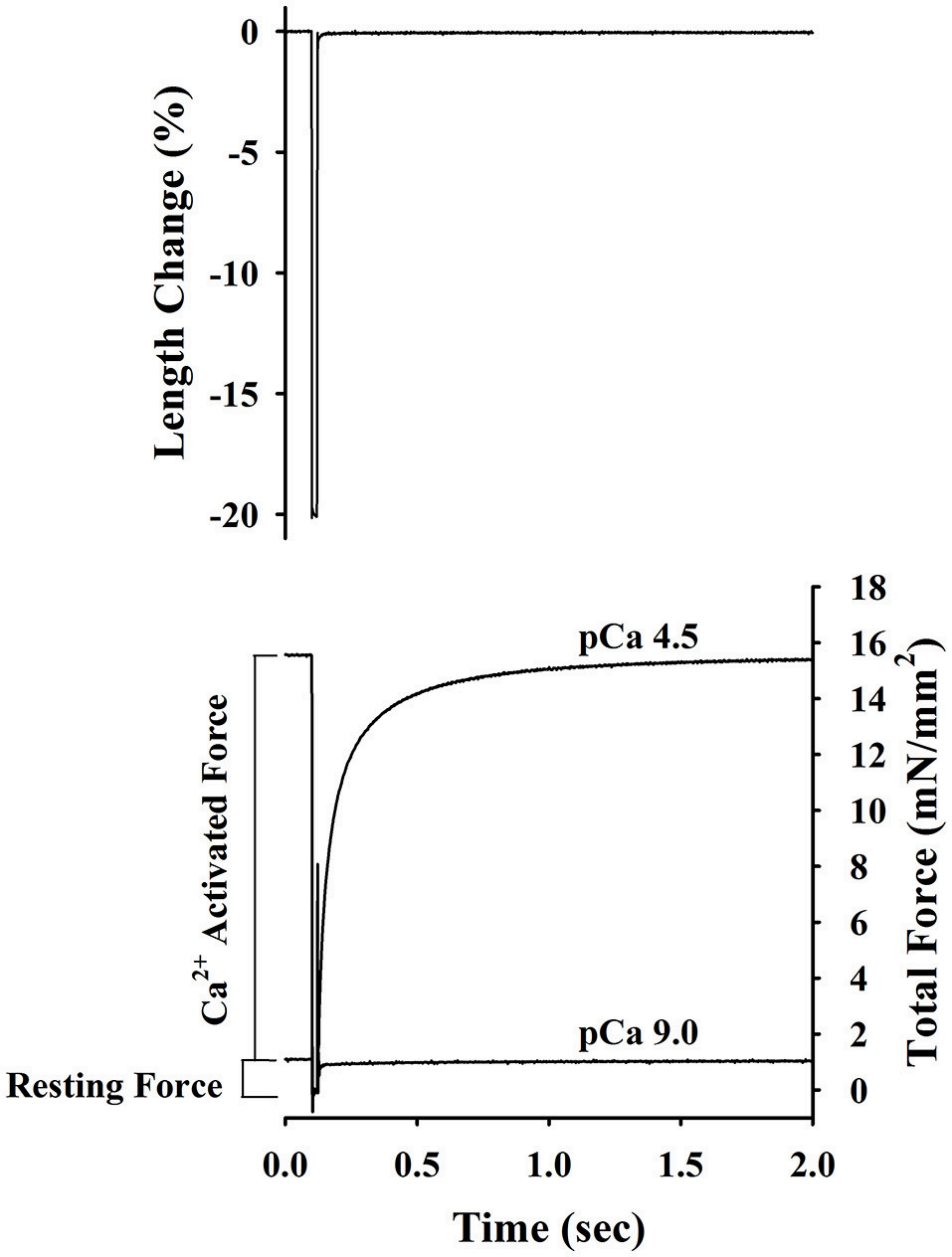
Experimental protocol for determining of passive force, Ca^2+^-activatedforce and rate constant of force redevelopment (*k*_tr_) in rat skinned right ventricular trabecula. The bottom panel shows the changes in force recorded before, during and after a step change in length (top panel) of a rat skinned right ventricular trabecula. Once active force reached a steady state in pCa 4.5 and 9.0 solutions, muscle length was rapidly slackened by 20%, held at this length for 20 ms and finally re-stretched back to its original length. The Ca^2+^-activated force was determined by subtracting the resting force measured at pCa 9.0 from the total force measured at pCa 4.5. *k*_tr_ is the apparent rate constant of force redevelopment following re-stretch of trabeculae to its original length.

Passive force, active force-pCa, and *k*_tr_-pCa/force relationships were established at a mean SL of ~2.2 μm as described previously (Olsson et al., 2004; Patel et al., 2012). Briefly, the skinned trabeculae were stretched to a mean SL ~2.2 μm and after measuring length and width, the preparations were transferred first to pre-activating solution, then to Ca^2+^ activating solution, and finally back to pCa 9.0 solution. Once in Ca^2+^ activated solution, steady-state force and the apparent rate constant of force redevelopment (*k*_tr_) was measured simultaneously using the modified multi-step protocol developed by Brenner and Eisenberg (1986) as described in detail previously (Patel et al., 2001) and illustrated in Figure 1. Briefly, after force reached a steady level in activating solution (pCa 6.2–4.5), the length of the preparation was rapidly reduced by ~20%, held for ~20 ms, and then re-stretched back to its original length. As a result, there was an initial transient increase, followed by a decrease in force (seen as a spike in the force trace) and subsequent slower recovery of force to near the initial steady-state level. *k*_tr_ reported in the present study is the rate constant of force redevelopment after the spike. The drop in force recorded in solution of pCa 9.0 was considered to be passive force and was therefore subtracted from the drop in total force at each pCa to yield Ca^2+^ activated force (P). The protocol was repeated to establish active force-pCa and *k*_tr_-pCa/relative active force relationships. After completing mechanical measurements, the trabeculae were detached from the points of attachment, placed in sodium dodecyl sulfate (SDS) sample buffer (8M Urea, 2 M thiourea, 0.05 M Tris pH6.8, 75 mM DTT, 3% SDS, and 0.01% bromophenol blue) and stored at −80°C until subsequent protein analysis.

### SDS page silver stain protein content analysis

To examine the expression profile of MHC isoforms, samples were prepared using RV free wall isolated from normoxic and hyperoxic treated rats and gels were prepared as described previously (Warren and Greaser, 2003). Briefly, RV free wall was homogenized in relaxing solution and homogenate washed with fresh relaxing solution. Next, the homogenate was incubated for 30 min in relaxing solution containing 1% Triton-100. After the incubation period, the homogenate was washed three times with relaxing solution and 2.4 mg (wet weight) of homogenate was suspended in 100 μL SDS sample buffer and stored at −80°C until subsequent protein analysis. A 17 mL solution of resolving gel (6%T and 2% C) was prepared by mixing 8.27 mL water, 1.7 ml 50% glycerol (v/v), 2.55 mL 40% acrylamide (37.5:1 cross-linked with DATD), 4.24 mL 1.5 M Tris, pH 8.8, 170 μL 10% SDS (w/v), 50 μL 10% ammonium persulfate (w/v), and 20 μL TEMED. The resolving gel was poured into an empty BioRad Criterion cassette and water was added to the top of the resolving gel to form a flat surface. After an hour of polymerization, the gel was stored in the cold room overnight. Next day, water was drained out and stacking gel (3%T and 1.5%C; 1.15 mL water, 1 ml 50% glycerol (v/v), 1.5 mL 10% acrylamide (5.6:1 cross-linked with DATD), 1.3 mL 0.5 M Tris, pH 6.8, 50 μL 10% SDS (w/v), 30 μL 10% ammonium persulfate (w/v), and 25 μL TEMED) was poured over the resolving gel. A 12 well comb was inserted and stacking gel was allowed to polymerize for an hour. The gel cassette was then inserted into a BioRad Criterion gel box pre-filled with ice cold lower running buffer (25.09 mM Tris-base, 19.98 mM glycine, 3.47 mM SDS, and 2 mM 2-mecaptoethanol). The comb was removed, wells were washed with water and the chamber was filled with ice cold upper running buffer (50.18 mM Tris-base, 39.96 mM glycine, 6.94 mM SDS, and 10 mM 2-mecaptoethanol). The samples were defrosted and 4 μL of sample was added to 36 μL of sample buffer. The diluted sample was heated (95°C) for 3 min and allowed to cool down before loading 10 μL on to the gel. Electrophoresis was done at 16 mA constant current for 4 h in the cold room. At the end of electrophoresis, the gel was removed from the cassette and silver stained using method described previously (Shevchenko et al., 1996) with following modification (Stelzer et al., 2006). The gels were (a) incubated overnight in fixing solution containing 50% methanol and 10% acetic acid, (b) washed for 20 min (4x ddH_2_O changes) with ddH_2_O, (c) incubated for 1.5 min in 0.01% sodium thiosulfate solution and then rinsed 4x with ddH_2_O, (d) incubated for 20 min in 0.09% silver nitrate solution and then rinsed 4x with ddH_2_O, (e) incubated in developing solution containing 0.0004% sodium thiosulfate, 2% potassium carbonate, and 0.0068% formaldehyde until protein bands were visible and then rinsed 4x with ddH_2_O, (f) incubated for 20 min in destaining solution containing 10% methanol and 10% acetic acid, and (g) finally washed for 30 min (6x ddH_2_O changes) with ddH_2_O.

To examine expression profile of myofibrillar proteins, the normoxic and hyperoxic trabeculae stored in SDS sample buffer were electrophoresed using 12% Tris-HCl Precast Criterion gels (BioRad, Hercules, CA). The gel cassette was inserted into BioRad Criterion gel box pre-filled with running buffer (25.09 mM Tris-base, 19.98 mM glycine, 3.47 mM SDS). The comb was removed, wells were washed with water and the chamber was filled with running buffer. The samples were defrosted, sonicated for 15 min and 8 μL of sample was loaded on to the gel. Electrophoresis was done at constant volts (150 V) for 1.5 h at room temperature. At the end of the run, the gels were removed from the cassette and silver stained as described above. Both gels were imaged and protein bands quantified using BioRad Chemi Doc MP Imaging System (BioRad, Hercules, CA).

### Data analysis and statistics

Cross-sectional areas of skinned trabeculae were calculated by assuming that the trabeculae were cylindrical and by equating the width, measured from video images of the mounted preparations, to diameter. Each Ca^2+^ activated force (P) at pCa between 6.2 and 5.4 was expressed as a fraction of the maximum Ca^2+^ activated force (P_o_) developed by the same preparations at pCa 4.5, i.e., P/P_o_. To determine the Ca^2+^ sensitivity of isometric force (pCa_50_), force-pCa data were fitted with the Hill equation: P/P_o_ = [Ca^2+^]^n^/(*k*^n^ + [Ca^2+^]^n^)], where n is the slope (Hill coefficient) and *k* is the Ca^2+^ concentration for half-maximal activation (pCa_50_). *k*_tr_ was determined by linear transformation of the half-time of force recovery (*k*_tr_ = −ln 0.5 × (t_1/2_)^−1^), as described previously (Chase et al., 1994; Patel et al., 2001). All data are presented as means ± standard error (SE). Statistical analysis of the RV pressure data was performed using two-way ANOVA (age and group main effects; GraphPad Prism 6;GraphPad Software Inc., San Diego, CA), all other analysis with unpaired *t*-tests (Sigma Plot 11.Ink; Systat Software Inc., San Jose, CA), *p*-values <0.05 were taken as indicating significant differences.

## Results

### Recovery of RV pressure from day 21 to day 35 in hyperoxia exposed rats

**Figure 2:**
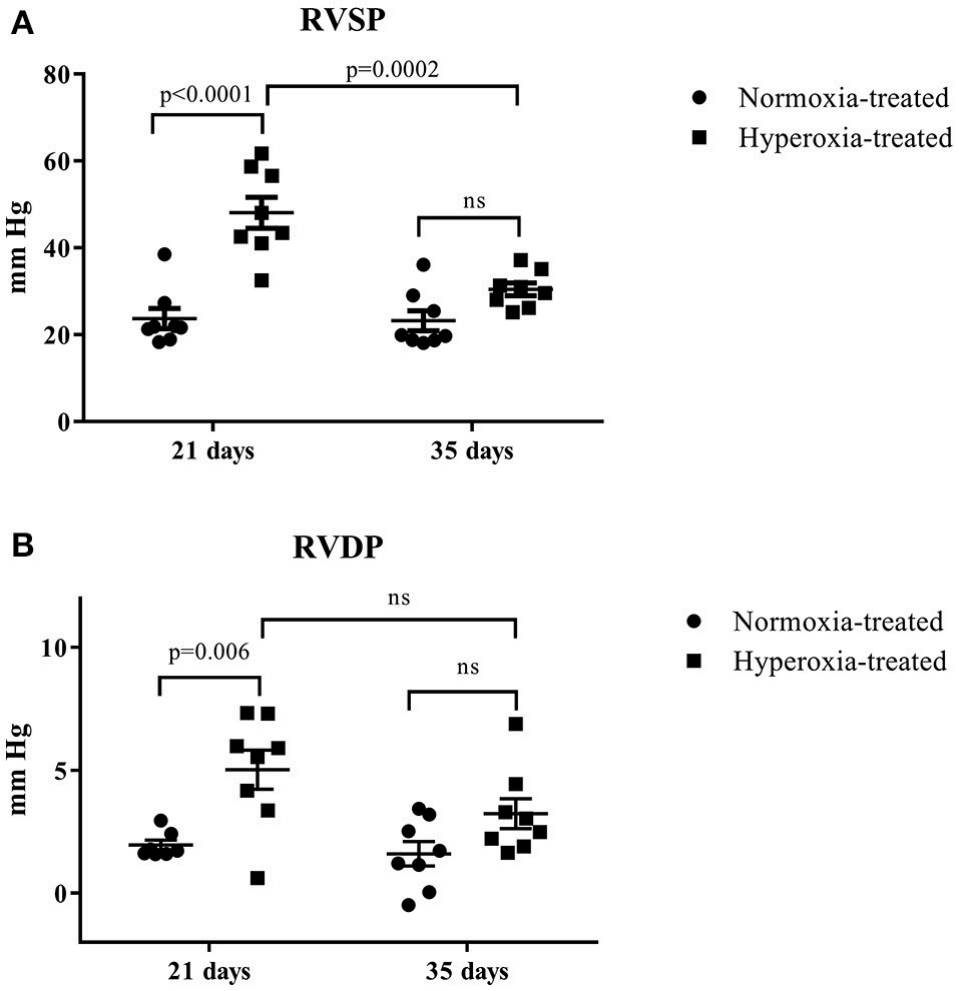
Effect of neonatal exposure to hyperoxia on development of pulmonary hypertension. At postnatal day 21 and day 35, RV systolic pressure (RVSP) **(A)** and RV diastolic pressure (RVDP) **(B)** were determined. The cross line shows the mean and the SE (*n* = 8 hearts). Statistical analysis was done using two-way ANOVA with Tukey's multiple comparisons test. *p*-values < 0.05 were considered significant.

A total of 49 rats were exposed to postnatal normoxia, and 40 rats were exposed to postnatal hyperoxia. Sixteen normoxia and hyperoxia exposed rats were used for the determination of RV pressures at 21 and 35 days of age (*n* = 8 in each group), respectively. There was a significant difference (*p* < 0.05) in body weight at day 21 (51.4 ± 1.4 vs. 42.4 ± 1.6 g) but no difference in body weight at day 35 (127.0 ± 4.7 vs. 117.1 ± 3.2 g) in normoxia treated vs. hyperoxia treated rats, respectively. The systolic and diastolic RV pressure (Figure 2) was significantly higher in the hyperoxic group at day 21 compared to the normoxic group at the same time point. This difference between the normoxic and hyperoxic group resolved by day 35 with RV pressure in the hyperoxic group being significantly lower compared to day 21. This suggests a recovery of the hyperoxia-induced PH seen at day 21.

### Effects of hyperoxia exposure on passive force, maximum Ca^2+^ activated force, apparent cooperativity in activation of force (n_H_), and Ca^2+^ sensitivity of force (pCa_50_)

**Figure 3:**
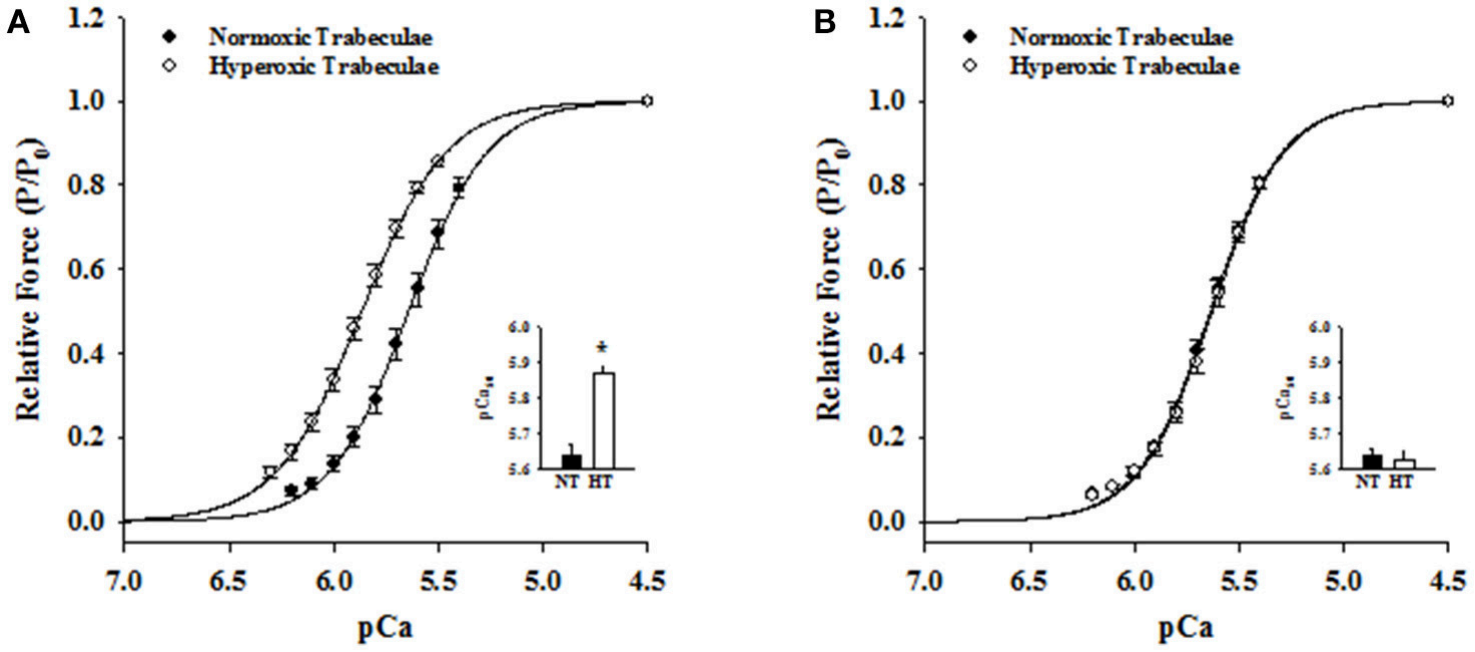
Effects of neonatal exposure to hyperoxia on apparent cooperativity in the activation of force (n_H_) and the Ca^2+^ sensitivity of force (pCa_50_) in 21- and 35-day skinned right ventricular trabeculae. Force-pCa relationships were measured in 21-Day normoxic (closed circles; *n* = 18 trabeculae from 15 hearts) and hyperoxic (open circles; 16 trabeculae from 12 hearts) trabeculae **(A)** and 35-Day normoxic (closed circles; *n* = 15 trabeculae from 8 hearts) and hyperoxic (open circles; 9 trabeculae from 8 hearts) trabeculae **(B)**. Submaximal Ca^2+^activated forces at pCa 6.2–5.4 were expressed as a fraction of maximum Ca^2+^activated force at pCa 4.5. The solid lines were generated by fitting the mean data with the Hill equation described in Methods and Materials. The respective fitted values of n_H_, apparent cooperativity of activation of force, and pCa_50_, an index for Ca^2+^ sensitivity of force, for 21-day normoxic trabeculae were 2.29 and pCa 5.64 and for hyperoxic trabeculae were 2.13 and pCa 5.87 **(A)**. The respective fitted values of n_H_ and pCa_50_ for 35-day normoxic trabeculae were 2.51 and pCa 5.64 and for hyperoxic trabeculae were 2.51 and pCa 5.63 **(B)**. Inset shows the pCa_50_ values determined in normoxic (black bars) and hyperoxic (white bars) trabeculae. Data points are the means ± SE. ^*^Significantly different (*p* < 0.05) from values determined in normoxic trabeculae.

Steady-state mechanical properties were assessed using right ventricular trabeculae isolated from a second cohort of 21-day normoxic (*n* = 18 trabeculae/15 rats) and hyperoxic (*n* = 16 trabeculae/12 rats) and 35-day normoxic (*n* = 15 trabeculae/8 rats) and hyperoxic (*n* = 9 trabeculae/8 rats) (Table 1). At pCa 9.0, passive force generated by 21-day hyperoxic trabeculae was almost twice (*p* < 0.001) than age matched normoxic trabeculae, and by 35-day hyperoxic trabeculae generated similar amount of passive force as age matched normoxic trabeculae (Table 1). At pCa 4.5, maximum Ca^2+^ activated force generated by 21-day hyperoxic trabeculae was ~70% greater (*p* = 0.002) than age matched normoxic trabeculae, and by 35-day hyperoxic trabeculae were similar to age matched normoxic trabeculae (Table 1). At sub-maximal Ca^2+^ (pCa 6.2–5.4), 21-day hyperoxic trabeculae also generated more force than age matched normoxic trabeculae, resulting in a left shift of hyperoxic trabeculae sigmodal force-pCa relationships compared to age matched normoxic trabeculae (Figure 3A). Fitting the force-pCa relationships with the Hill equation yielded significantly higher pCa_50_ values (ΔpCa_50_ = 0.23; *p* < 0.001), implying elevated Ca^2+^ sensitivity of force, and lower n_H_ values (Δ n_H_ = 0.3; *p* = 0.005), which implies depressed apparent cooperativity in activation of force, for hyperoxic compared to normoxic trabeculae. On the other hand, sub-maximal forces generated by 35-day hyperoxic trabeculae were similar to those measured in age matched normoxic trabeculae and as a result there was no discernable difference between the sigmodal force-pCa relationships established in hyperoxic and age matched normoxic trabeculae (Figure 3B). Fitting the force-pCa relationships with the Hill equation yielded similar pCa_50_ values and n_H_ values for hyperoxic and age matched normoxic trabeculae. These results suggest that the neonatal exposure to hyperoxia has profound early effects on steady-state force production and that these effects wane as the PH resolves.

**Table 1:** Summary of mechanical properties of skinned right ventricular trabeculae isolated from 21-and 35-day old Normic and Hyperoxic rat myocardium.

**Treatment**	**Passive force (mN/mm^2^)**	**Maximum Ca^2+^ activated force (mN/mm^2^)**	**Hill coefficient (n_H_)**	**Ca^2+^ sensitivity of force (pCa_50_)**	**Maximum rate of force redevelopment (*k*_tr,_s^−1^)**
**21-DAY OLD**					
Normic (18/15)	1.35 ± 0.18	13.16 ± 2.06	2.56 ± 0.07	5.64 ± 0.03	10.48 ±1.08
Hyperoxic (16/12)	2.69 ± 0.29^*^	22.05 ± 1.30^*^	2.26 ± 0.08^*^	5.87 ± 0.02^*^	8.28 ± 1.02
**35-DAY OLD**					
Normic (15/8)	1.83 ± 0.21	16.20 ± 1.59	2.60 ± 0.05	5.64 ± 0.02	11.74 ± 0.58
Hyperoxic (9/8)	1.44 ± 0.25	12.30 ± 1.56	2.58 ± 0.05	5.63 ± 0.02	10.43 ± 0.78

*All values are expressed as means ± SE, with number of skinned right ventricular trabeculae/rat hearts given in parentheses. At SL of 2.2 μm, passive force was measured at pCa 9.0. Maximum force and the rate constant of force redevelopment (k_tr_) were measured at pCa 4.5. pCa_50_ and n_H_ values were derived by fitting the force-pCa relationships to a Hill equation*.

* *Significantly different (p < 0.05) from values recorded in age matched normoxic skinned trabeculae*.

### Effects of hyperoxia on rate of force redevelopment (k_tr_)

**Figure 4:**
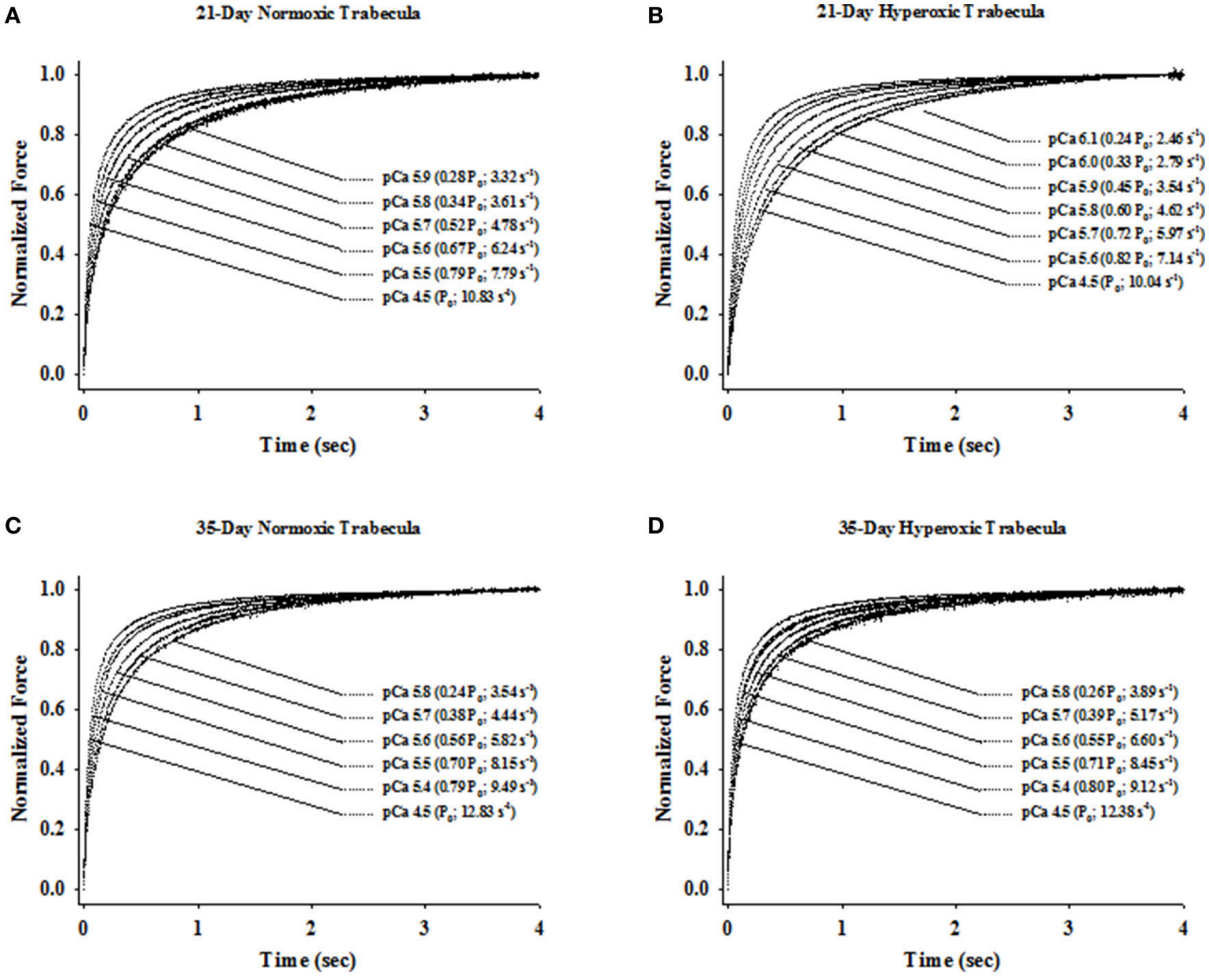
Ca^2+^- and force-dependent changes in apparent rate constant of force redevelopment (*k*_tr_) in 21- and 35-Day normoxic and hyperoxic skinned right ventricular trabecula. The rate of force redevelopment (*k*_tr_) was measured as described in Methods and Materials at sub-maximal (pCa 6.1–5.4) and maximum (pCa 4.5) [Ca^2+^]_free_ in skinned right ventricular trabecula isolated from 21-Day normoxic **(A)** and hyperoxic **(B)** and 35-Day normoxic **(C)** and hyperoxic **(D)** rat ventricle. The force transients were expressed relative to the peak steady state force attained after the step change in muscle length. Both, relative force and *k*_tr_ values for a given [Ca^2+^]_free_ is shown in parentheses.

**Figure 5:**
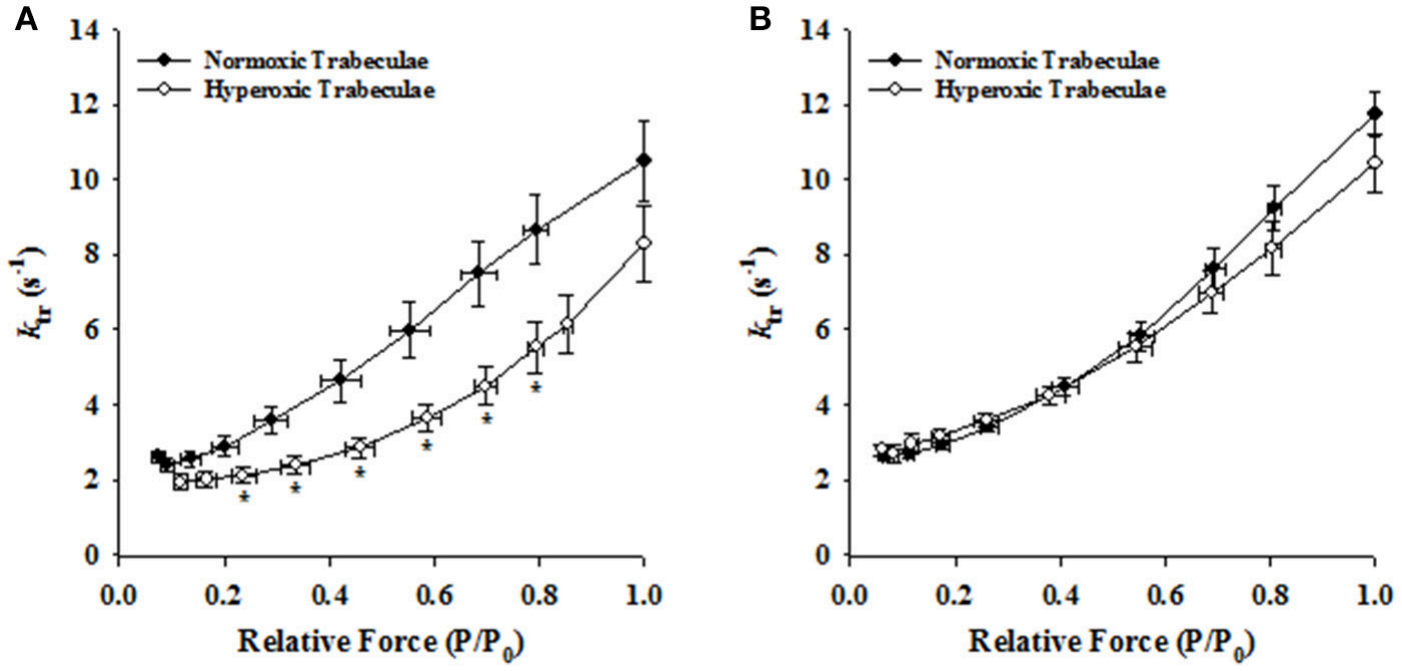
Effects of neonatal exposure to hyperoxia on the rate of force redevelopment (*k*_tr_) as a function of relative force (P/P_0_) relationships in 21- and 35-day skinned right ventricular trabeculae. Rate of force redevelopment (*k*_tr_)-relative force (P/P_0_) relationships were characterized in 21-Day normoxic (closed circles; *n* = 18 trabeculae from 15 hearts) and hyperoxic (open circles; 16 trabeculae from 12 hearts) trabeculae **(A)** and 35-Day normoxic (closed circles; *n* = 15 trabeculae from 8 hearts) and hyperoxic (open circles; 9 trabeculae from 8 hearts) trabeculae **(B)**. ^*^Significantly different (*p* < 0.05) from values determined in normoxic trabeculae.

Irrespective of age, both normoxic and hyperoxic trabeculae exhibited [Ca^2+^]_free_ (and force)-dependent changes in the rate of force redevelopment (*k*_tr_), confirming earlier results from rat (Wolff et al., 1995; Palmer and Kentish, 1998; Patel et al., 2012) myocardium. That is, increasing the [Ca^2+^]_free_ from pCa 6.2 to pCa 4.5 elevated the values of *k*_tr_ from 2.62 ± 0.14 to 10.48 ± 1.08 s^−1^ and 1.93 ± 0.21 to 8.28 ± 1.02 s^−1^ in 21-day normoxic and hyperoxic trabeculae and from 2.61 ± 0.12 to 11.74 ± 0.58 s^−1^ and 2.79 ± 0.17 to 10.43 ± 0.78 s^−1^ in 35-day normoxic and hyperoxic trabeculae. To illustrate this, records of force redevelopment at various levels of [Ca^2+^]_free_ are shown in Figure 4 for 21-day normoxic (Figure 4A) and hyperoxic (Figure 4B) and 35-day normoxic (Figure 4C) and hyperoxic (Figure 4D) trabecula, where steady state forces at each pCa were normalized to 1.0 to provide better visualization of variations in kinetics of force redevelopment. Figure 5 shows the curvilinear *k*_tr_-relative force relationships observed in 21-day (Figure 5A) and 35-day (Figure 5B) normoxic and hyperoxic trabeculae. At pCa 4.5, both 21 and 35-day hyperoxic trabeculae redeveloped maximum Ca^2+^ activated force at similar rates as age matched normoxic trabeculae. At sub-maximal free [Ca^2+^] (pCa 6.2–5.4), 21-day hyperoxic trabeculae redeveloped sub-maximal forces at significantly slower rates than age matched normoxic trabeculae and as a result the curvilinear *k*_tr_-relative force relationships established in hyperoxic trabeculae were to the right of those established in normoxic trabeculae (Figure 5A). On the other hand, 35-day hyperoxic trabeculae redeveloped sub-maximal forces at rates similar to age matched normoxic trabeculae and as a result there was no discernable difference between curvilinear *k*_tr_-relative force relationships established in hyperoxic and age matched normoxic trabeculae (Figure 5B). These results indicate that the neonatal exposure to hyperoxia has profound effects on cross-bridge cycling kinetics and that the effects are reversible in rat myocardium. These reversible effects are coincident with the normalization of RV pressure at 35 days.

### Effects of hyperoxia on myofibrillar protein expression

**Figure 6:**
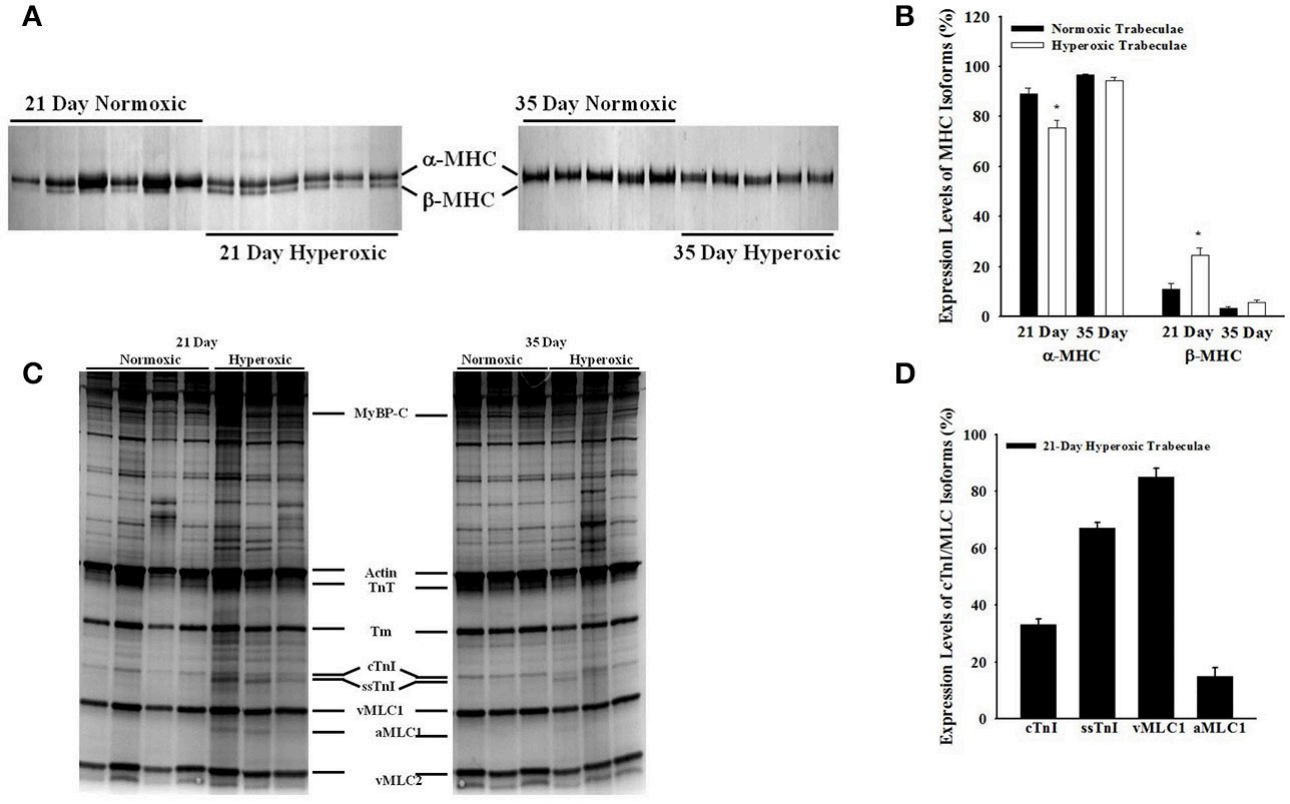
Effects of neonatal exposure to hyperoxia on myofibrillar protein compositions in 21- and 35-day skinned right ventricular trabeculae. A typical expression profile of myosin heavy chain isoforms (MHC) in 6 normoxic and hyperoxic 21-day right ventricles and 5 normoxic and hyperoxic 35-day right ventricles **(A)** and other myofilament proteins in four normoxic and hyperoxic 21-day skinned right ventricular trabeculae and 3 normoxic and hyperoxic 35-day skinned right ventricular trabeculae **(C)** examined by SDS-PAGE as described in Methods and Materials. Myofibrillar proteins are identified in order of increasing mobility. **(B)** 21-day hyperoxic myocardium was expressing significantly lower α-MHC and higher β-MHC than normoxic myocardium, whereas, 35-day hyperoxic and normoxic myocardium were expressing similar levels of both α- and β-MHC. **(D)** 21-day hyperoxic trabeculae were expressing both cTnI and ssTnI, and vMLC1 and a-MLC1 isoforms. Beside above differences, there were no other major detectable differences in the expression profiles of myofibrillar proteins between normoxic and hyperoxic trabeculae. cMyBP-C, cardiac myosin binding protein–C; TnT, troponin T; Tm, tropomyosin; cTnI, cardiac troponin I; ssTnI, slow skeletal Troponin I; vMLC1, ventricular myosin light chain 1; aMLC1, atrial myosin light chain 1; vMLC2, ventricular myosin light chain 2. ^*^Significantly different (*p* < 0.05) from values determined in normoxic right ventricular wall.

Figure 6 shows a typical SDS-PAGE analysis of MHC isoforms (Figures 6A,B) and myofibrillar protein expression (Figures 6C,D) in 21- and 35-day normoxic and hyperoxic myocardium. Figures 6A,B shows that 21-day hyperoxic RV expressed 14% less α-MHC and more β-MHC than aged matched normoxic RV. Whereas, 35-day hyperoxic RV expressed similar levels of both α-MHC and β-MHC as aged matched normoxic RV. It is also apparent from Figure 6C that both 21- and 35-day hyperoxic trabeculae expressed similar isoforms of key myofibrillar proteins as normoxic trabeculae (cardiac myosin binding protein C (cMyBP-C), actin, troponin T (TnT), tropomyosin (Tm), ventricular myosin light chain 1, and 2 (vMLC1 and vMLC2), respectively. While both 21 and 35-day normoxic trabeculae were found to express 100% cTnI, an observation consistent with a previous study which reported complete conversion of ssTnI to cTnI by 15 days after birth in rat (Warren et al., 2004), 21-day hyperoxic trabeculae expressed both cTnI (33 ± 2%) and ssTnI (67 ± 2%) (Figure 6D). With the exception of one hyperoxic trabeculae expressing both cTnI (31%) and ssTnI (69%), 35-day normoxic and hyperoxic rat trabeculae expressed predominately cTnI. In addition, 21-day, but not 35-day, hyperoxic trabeculae expressed aMLC1 (15 ± 3%) and vMLC1 (85 ± 3%) (Figure 6D), an observation consistent with PH-induced expression of aMLC1in neonatal porcine right ventricle (Morano et al., 1998). These results suggest that neonatal exposure to hyperoxia elevates expression of β-MHC, disrupts transition of ssTnI to cTnI, stimulates expression of aMLC1, and indicates that most of these effects are reversible in rat myocardium.

## Discussion

The goal of the present study was to use skinned right ventricular trabeculae isolated from hearts of 21 and 35-day old rats to examine the impact of neonatal hyperoxia exposure on contractile properties and protein expression within the RV in a model of BPD. We demonstrated for the first time that neonatal exposure to hyperoxia has profound effects on passive force (increase), maximum Ca^2+^ activated force (increase), Ca^2+^ sensitivity of force (increase), apparent cooperativity in activation of force (decrease), cross-bridge cycling kinetics (slower), expression of β-MHC (higher), aMLC1, and the developmental transition of ssTnI to cTnI (delayed). Furthermore, these effects of neonatal exposure to hyperoxia on steady-state force production, cross-bridge cycling kinetics and protein expression normalize as RV afterload and PH improve by 35 days of age. Together these changes result in a hypercontractile RV as a neonatal adaptive response to hyperoxia-induced PH.

Chronic lung disease of prematurity, or BPD, is frequently complicated by PH, which results in a significant increase in neonatal morbidity and mortality (Baker et al., 2014). We chose to use rats at the ages of 21 and 35-days postnatal life which corresponds with weaning (~6 months of age) and pre-pubertal human stages of life, respectively (Sengupta, 2013). A previous study reported that preterm infants with PH and BPD demonstrated a mortality of 38% during a median follow up of 10.9 months (Khemani et al., 2007). However, the majority of individuals born preterm with chronic lung disease have a normalization of RV function and PA pressure in later childhood despite persistently abnormal lung function (Joshi et al., 2014). Our data demonstrates a similar finding to humans in which altered RV function and RV pressure in early stages of development normalizes with further developmental age. Furthermore, these findings of mechanical disruption of the RV is coincident with PH at 21 days postnatal rat life corresponds with a time in life where humans born preterm diagnosed with BPD and PH are most susceptible to higher mortality rates.

### Impact of hyperoxia on steady-state contractile properties of right ventricle

The 21-day hyperoxic trabeculae generated twice as much force as normoxic trabeculae (Table 1), an effect similar to the PH-induced increase in maximum Ca^2+^ activated force observed in the adult human (Rain et al., 2013), rat (Kogler et al., 2003), and mice (unpublished observation). While these results are consistent with the idea that higher density of thick and thin filaments allows 21-day hyperoxic trabeculae to generate more force, the finding of similar amount of maximum Ca^2+^ activated forces in 35-day hyperoxic and normoxic trabeculae is inconsistent with this mechanism. Previous studies reported that myocardium expressing ssTnI generates a maximum Ca^2+^ activated force similar to myocardium expressing cTnI (Fentzke et al., 1999; Arteaga et al., 2000; Konhilas et al., 2003; Ford and Chandra, 2012) whereas expression of aMLC1 results in a higher maximum Ca^2+^ activated force than expression of vMLC1 (Morano et al., 1998). Interestingly, our findings of increased maximum Ca^2+^activated force and aMLC1 in 21-day old hyperoxia exposed rats was associated with PH, which is similar to a report in a porcine model of PH (Morano et al., 1998). Taken together, the earlier observation of increased maximum Ca^2+^activated force in 21-day hyperoxic trabeculae and the similar amount of maximum Ca^2+^activated force generated by 35-day hyperoxic trabeculae compared to normoxic trabeculae suggests that expression of aMLC1 is likely to play a prominent role in increasing maximum Ca^2+^activated force in 21-day hyperoxic trabeculae.

At sub-maximal Ca^2+^, 21-day hyperoxic trabeculae generated more force than age matched normoxic trabeculae and as a result, the force-pCa relationships established in hyperoxic trabeculae were left-shifted by ~0.23 pCa units compared to those in normoxic trabeculae (Figure 3A), which corresponds to similar changes found in monocrotaline-induced PH (Kogler et al., 2003). In cardiac muscle, expression of either ssTnI (Fentzke et al., 1999; Arteaga et al., 2000; Konhilas et al., 2003; Ford and Chandra, 2012) or aMLC1 (Morano et al., 1997; Diffee and Nagle, 2003; Diffee, 2004) are known to shift force-pCa relationships to the left. Since 21-day hyperoxic trabeculae expressed both ssTnI and aMLC1, it is difficult to say with certainty that the increased Ca^2+^ sensitivity of force in 21-day hyperoxic trabeculae was due to the presence of ssTnI or aMLC1, or both. However, the finding of similar Ca^2+^ sensitivities of force in 35-day hyperoxic trabeculae and age-matched normoxic trabeculae (Figure 3B), in which there is no aMLC1 present and predominant expression of cTnI (Figure 6C), suggest that the hyperoxia-induced changes in expression of MLC1/TnI isoforms at 21 days is reversible and may be responsible for the increase in Ca^2+^ sensitivity in hyperoxic myocardium at this age. Taken together, our findings of increased maximum Ca^2+^activated force, Ca^2+^ sensitivity of force and changes in expression of MLC1/TnI isoforms at 21 days of age in our hyperoxia exposed rats are likely an adaptive response to PH. This is highlighted by the findings at 35 days of age where there were no differences in RV pressure, RV myofibrillar isoform expression and contractile properties.

### Impact of hyperoxia on dynamic contractile properties of right ventricle

In normoxic trabeculae, the rate of force redevelopment (*k*_tr_) varied with the level of activating [Ca^2+^]_free_ (or force), increasing as [Ca^2+^]_free_ (or force) was elevated from sub-maximal to maximal levels (Figures 4, 5). These Ca^2+^- and force-dependent changes in *k*_tr_ in normoxic trabeculae are consistent with previous results from rat (Wolff et al., 1995; Palmer and Kentish, 1998; Olsson et al., 2004; Patel et al., 2012), mice (Edes et al., 2007; Colson et al., 2012; Ford and Chandra, 2012), porcine (Edes et al., 2007) and human (Edes et al., 2007) myocardium. Both, 35-day normoxic and hyperoxic trabeculae also exhibited similar Ca^2+^- and force-dependent changes in *k*_tr_, suggesting no remaining significant effects of hyperoxia on these relationships. However, 21-day hyperoxic trabeculae redeveloped sub-maximal forces at a slower rate than age-matched normoxic trabeculae (Figure 4). Thus, when *k*_tr_ values were plotted against force normalized to maximum force, *k*_tr_-force relationships in hyperoxic trabeculae were right-shifted compared to those in normoxic trabeculae (Figure 5A), i.e., at equivalent forces, *k*_tr_ values were lower in hyperoxic trabeculae. In adult rat myocardium, a decrease in expression of α-MHC, and concomitant increase in expression of β-MHC, is known to slow the rate of force redevelopment (Fitzsimons et al., 1999; Rundell et al., 2005; Locher et al., 2011) and rate of relaxation (Fitzsimons et al., 1998). Thus, the possibility of the depressed cross-bridge cycling kinetics in 21-day hyperoxic trabeculae may be exclusively or in part due to a decrease in expression of α-MHC and concomitant increase in expression of β-MHC (Figure 6B). Previous studies have reported that expression of ssTnI has no significant effects on the rate of force redevelopment in skinned preparations (Ford and Chandra, 2012), whereas expression of aMLC1 increases contraction and relaxation time in whole heart (Morano et al., 1996, 1998; Fewell et al., 1998; Abdelaziz et al., 2004). Since there is expression of aMLC1 and ssTnI in 21-day hyperoxic myocardium but not in normoxic myocardium, both aMLC1and ssTnI appears to be responsible for slower contraction kinetics in hyperoxic myocardium at this age.

Alternatively, the ability of hyperoxic trabeculae to generate more force than normoxic trabeculae can be explained in the context of a two-state kinetic model of cross-bridge interaction proposed by Huxley (1957) and modified by Brady (1991). In this model, multiple states of the cross-bridge kinetic scheme are reduced to just two states, i.e., the transition from non-force-generating to force-generating states is described by *f*_*app*_, whereas *g*_*app*_ describes the transition from the force-generating state back to the non-force-generating state. Steady isometric force (P) is then equal to *N* × *F* × [*f*_*app*_/(*f*_*app*_ + *g*_*app*_)], where *N* is the number of cycling cross-bridges, *F* is the average force per cross-bridge and *k*_tr_ = *f*_app_ + *g*_app_. Thus, the hyperoxia-induced increase in Ca^2+^ sensitivity of force in the present study may be due to an increase in *N*, or *F*, or the proportion of cross-bridges in the force generating state as a result of an increase in *f*_*app*_, a decrease *g*_*app*_, or both. An increase in either the probability of myosin cross-bridge binding to actin (in case of aMLC1; Schaub et al., 1998) or the binding affinity of TnC for Ca^2+^ (in case of ssTnI) would increase N and facilitate cooperative binding of myosin cross-bridges to actin. The latter would be manifested as a decrease in n_H_ (an index of apparent cooperativity in activation of force; Table 1) of the force-pCa relationship, and a decreased cross-bridge detachment rate (*g*_*app*_), which would be manifested as decrease in *k*_tr_. Interestingly, *g*_*app*_ derived from natural logarithm of *k*_tr_-relative force relationship (data not shown) was lower in 21-day hyperoxic (1.44 s^−1^) than normoxic (2.17 s^−1^) trabeculae. Thus, it appears that the combined effect of β-MHC, ssTnI (increased binding affinity of TnC for Ca^2+^) and aMLC1 (increased probability of myosin cross-bridge binding to actin) are important for the reduced kinetics in the RV at 21 days of age. Although, no previous studies have explored cross-bridge cycling kinetics in a model of BPD, we can infer that reduced cross-bridge cycling kinetics in 21 day hyperoxia exposed rats is likely due to the adaptive response to pulmonary pressure overload and the subsequent myofibrillar isoform expression changes at 21 days of age in this model.

## Conclusion

In summary, we found hyperoxia-induced changes in expression of MHC, TnI, and MLC1 isoforms are reversible upon normalization of RV pressure and responsible for altering both steady-state force production and cross-bridge cycling kinetics in rat myocardium. Increasing developmental age in this rodent model of BPD is associated with a reversal of PH-induced RV dysfunction, which has also been observed in humans with BPD associated with preterm birth. This work underscores the importance of reducing RV afterload to allow for recovery of RV function in both animal models and humans with BPD.

## Author contributions

JP: Designed and conducted experiments, analyzed data and wrote the manuscript; GB: analyzed data, wrote and edited the manuscript; RB: Conducted experiments and edited the manuscript; KG: Contributed to design, wrote and edited the manuscript; KH: Conducted experiments; AH: Conducted experiments; GD: edited the manuscript; TH: Conducted experiments, analyzed data and edited the manuscript; RM: Oversaw experiments, wrote and edited the manuscript; ME: Contributed to design, oversaw experiments, wrote and edited the manuscript.

### Conflict of interest statement

The authors declare that the research was conducted in the absence of any commercial or financial relationships that could be construed as a potential conflict of interest.
